# Nitrogen-rich energetic polymer powered aluminum particles with enhanced reactivity and energy content

**DOI:** 10.1038/s41598-022-12949-0

**Published:** 2022-05-25

**Authors:** Yaru Li, Hui Ren, Xinzhou Wu, Huixin Wang, Xilong Yu

**Affiliations:** 1 State Key Laboratory of Explosion Science and Technology, Beijing Institute of Technology, Beijing, 100081 China; 2 State Key Laboratory of High Temperature Gas Dynamics, Institute of Mechanics, Chinese Academy of Sciences, Beijing, 100190 China

**Keywords:** Energy science and technology, Materials science

## Abstract

Aluminum particles are of significant interest in enhancing the energy release performance of explosives. One of the major impediments to their use is that Al_2_O_3_ shell significantly decreases overall performance. To address this issue, we investigate creating aluminum particles with a glycidyl azide polymer (GAP) coating to improve their reactivity while retaining their energy content. We found that the aluminum particles were coated with a GAP layer of thickness around 8.5 nm. The coated aluminum particles were compared to non-coated powder by the corresponding reactivity parameters obtained from simultaneous differential scanning calorimetry, thermal gravimetric analysis, coupled with mass spectral and infrared spectral analyses. Besides, the comparison on the energy content was also conducted based on *P*–*t* tests and a laser-induced air shock from energetic materials (LASEM) technique. It was found that GAP shifted the oxidation onset of aluminum particles to a lower temperature by ~ 10 °C. Besides, the oxidation activation energy of aluminum particles was also reduced by ~ 15 kJ mol^−1^. In return, aluminum particles reduced the activation energy of the second stage decomposition of the GAP by 276 kJ mol^−1^. And due to the synergistic effect between aluminum and GAP, the decomposition products of GAP were prone to be oxycarbide species rather than carbonitride species. In addition, the *P*–*t* test showed the peak pressure and pressurization rate of GAP coated aluminum particles were separately 1.4 times and 1.9 times as large as those of non-coated aluminum particles. Furthermore, the LASEM experiment suggested the shock wave velocity of the GAP coated aluminum particles was larger than that of non-coated aluminum particles, and the largest velocity difference for them could be 0.6 km s^−1^. This study suggests after coating by GAP, the aluminum particles possess enhanced reaction performance, which shows potential application value in the fields of aluminized explosives and other energetic fields.

## Introduction

Aluminum (Al) powders can effectively enhance the energy content and regulate the reaction performance of energetic materials, owing to its high chemical activity and energy density^1–4^. However, these excellent properties have not been fully exploited because of the inherent oxide shell, especially in the case of Al nanoparticles^5–8^. The thick oxide shell can significantly retard mass and heat transfer to the Al core, which leads to low reaction efficiency^9–12^. To address this issue, one prevailing strategy is to modify the Al surface with polymer layer to reduce the content of the oxide shell^13–15^. This strategy may improve the reactivity of the Al particles, however, it will decrease the energy content of the system due to the inert nature of modified materials.

As an energetic binder, glycidyl azide polymer (GAP) has a high density of 1.3 g cm^−3^, good thermal stability, a positive enthalpy of formation of 117.2 kcal mol^−1^, and a high burning rate^16–18^. It is one of the most promising alternatives to inert hydroxyl-terminated polybutadiene when it comes to improving the specific impulse of propellants and energy of the explosives^19^. In addition to the high reactivity, the GAP also shows good compatibility with energetic materials including propellants, explosives and Al particles, which are important for long-term storage safety^16,20–23^. It is therefore believed that the GAP has the potential to improve the reactivity of the Al particles while retaining their energy content. Accordingly, several studies have reported chemical or physical coating processes with the GAP^24,25^. However, the Al nanoparticles used in the studies are commercial ones which have gone through passivation process to get thick oxide shell. Besides, the coating processes involve complex chemical reactions which require additional chemical agents to bond between the GAP and the oxide shell. Therefore, it would further jeopardize the content of the active Al particles. Furthermore, those additional chemical agents used in the coating processes are not environmental friendly. And the complex chemical reactions also make the coating methods not suitable for mass production.

To enable the wide application of the GAP coated Al particles, it is important for the preparation process to be simple and environmental friendly. Accordingly, we report a facile in-situ coating approach to prepare the GAP coated Al (ALG) particles. To retain a high content of the active Al in the resulted products, fresh Al nanoparticles produced by electrical explosion of wire are used and are carefully protected by the inert gas throughout the entire process. In addition, the GAP is coated on the Al nanoparticles with the help of physical adsorption force. Therefore, no additional chemical reactions or chemical agents are involved in the process. The resulted samples are characterized with respect to morphologies, constitutions, thermal analyses, and reaction performances under fast heating and laser stimuli. It is surprisingly found that the GAP could improve the reaction performances under both slow heating and fast heating/laser stimuli. The mechanisms for the improvements are discussed.

## Results and discussion

### Morphology and constitution

**Figure 1:**
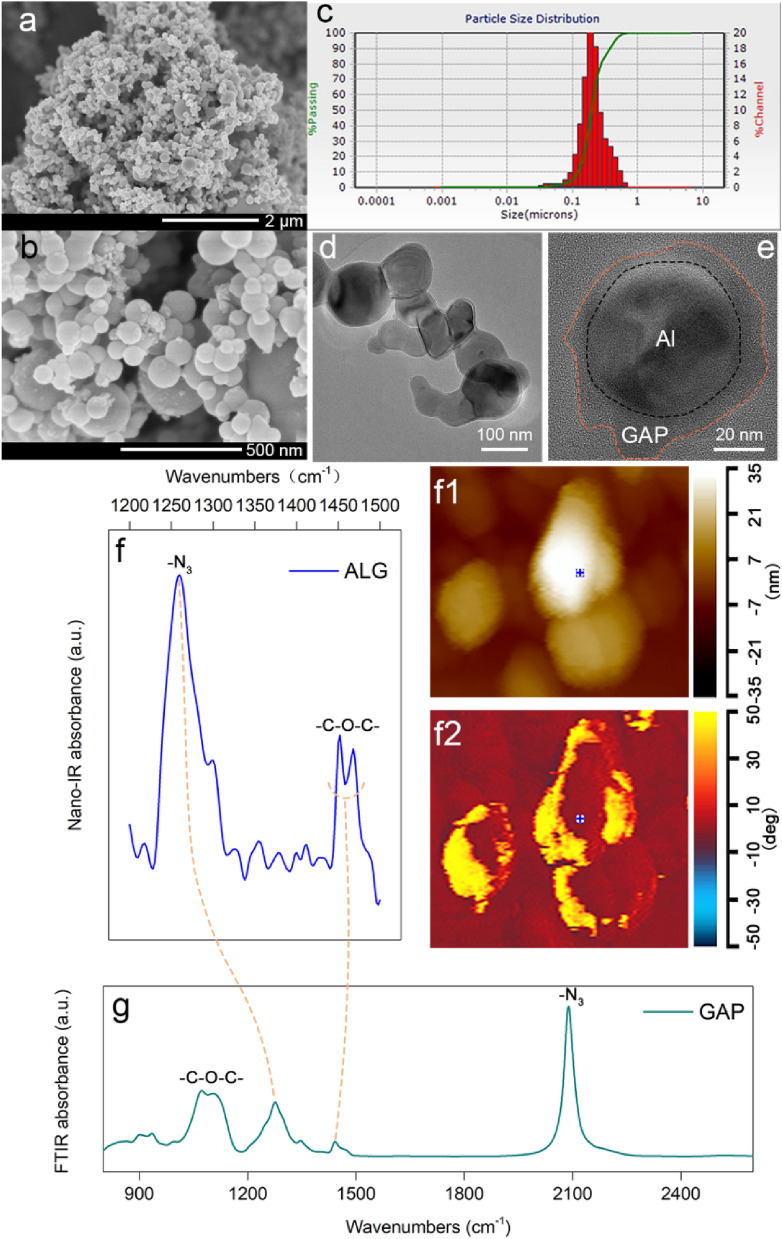
Scanning electron microscopy (SEM), transmission electron microscopy(TEM), nano-IR and FTIR spectra of the ALG. (**a**,**b**) are the SEM images; (**c**) is laser grain size distribution; (**d**,**e**) are the TEM images; (**f**) is the corresponding nano-IR spectrum of the blue spot in heigh image (**f1**) and phase image (**f2**); (**g**) the FTIR spectrum of the GAP.

Morphologies of the ALG particles were characterized by SEM and TEM, as shown in Fig. 1a,b. The particles were in the spherical shape with particle sizes continuously ranging from 50 nm to around 500 nm. Besides, the grain size distribution of the ALG particles in Fig. 1c was consistent with their size range in Fig. 1b, which showed the good stability of the particles. The resulted particles owned good stability due to the intense connection between the GAP and Al particles. The TEM images showed the GAP coating layers were in amorphous shapes (Fig. 1d). And the coating layer was uniformly distributed except one little bulge (Fig. 1e). The thickness of the coating layer, measured by the Image-Pro software, was around 8.5 ± 1.5 nm except the bulge whose thickness was 17 nm. Furthermore, in order to directly identify the GAP distribution, the nano-IR system was employed to in-situ characterize the constitution of the ALG surface. The results are shown in Fig. 1f,f1,f2. And Fig. 1f1,f2 were the corresponding height image and phase image of the Fig. 1f, respectively. As shown in Fig. 1f1, the thickest part of the particle was of around 35 nm. There were two distinct phase states on the sample surface in Fig. 1f2, the light colored part and the dark colored part separately corresponded to the GAP and the Al particle. The nano-IR spectrum of the blue spot on the surface in Fig. 1f1,f2 is shown in Fig. 1f. Through comparing with the FTIR spectrum of the GAP in Fig. 1g, it could be confirmed that the surface coating was the GAP. As the characteristic bonds of the GAP: C–O–C bond and C–N_3_ bond at wavenumbers around 1450 cm^−1^, 1270 cm^−1^ were both detected, respectively^26–29^.

### Non-isothermal reaction kinetics

**Figure 2:**
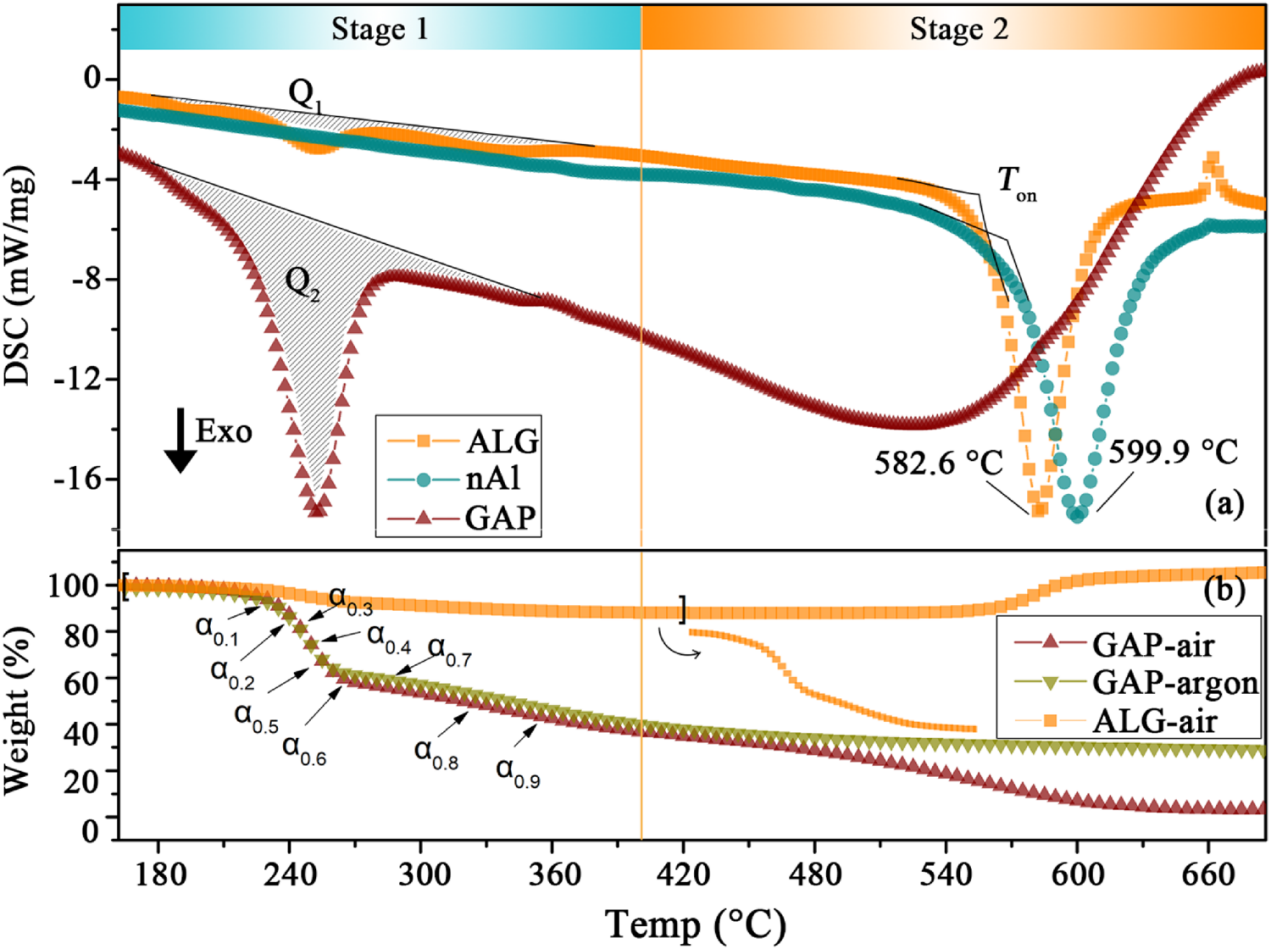
DSC thermographs of samples in air atmosphere at heating rate of 10 °C min^−1^ (**a**), TGA thermographs of GAP in air and argon atmosphere, and of ALG in air atmosphere at heating rate of 10 °C min^−1^ (**b**).

The DSC thermographs of ALG, GAP and nAl in air atmosphere at heating rate of 10 °C min^−1^ are shown in Fig. 2a. According to the DSC thermographs of the GAP and the nAl particles, the DSC thermograph of the ALG particles could be divided into two main exothermic processes. The first process ranged from 165 to 404 °C with a heat release (*Q*_1_) of 396.1 J g^−1^ and a weight loss of 11.24%. Under the same temperature range and atmosphere, the GAP experienced its first exothermic process with a heat release (*Q*_2_) of 2330 J g^−1^. Besides, the *Q*_1_ divided by the *Q*_2_ was equal to 16.9%, which was within the margin of error in accord with the weight ratio (16.7%) of the GAP in the ALG particles. In addition, according to the TGA thermographs of GAP in Fig. 2b, the weight loss of the GAP in air was the same with that of the GAP in argon over the temperature range of this process. Based on the above analyses, we could reach the conclusion that the first exothermic process of the ALG particles was caused by the decomposition of the GAP^30^.

**Figure 3:**
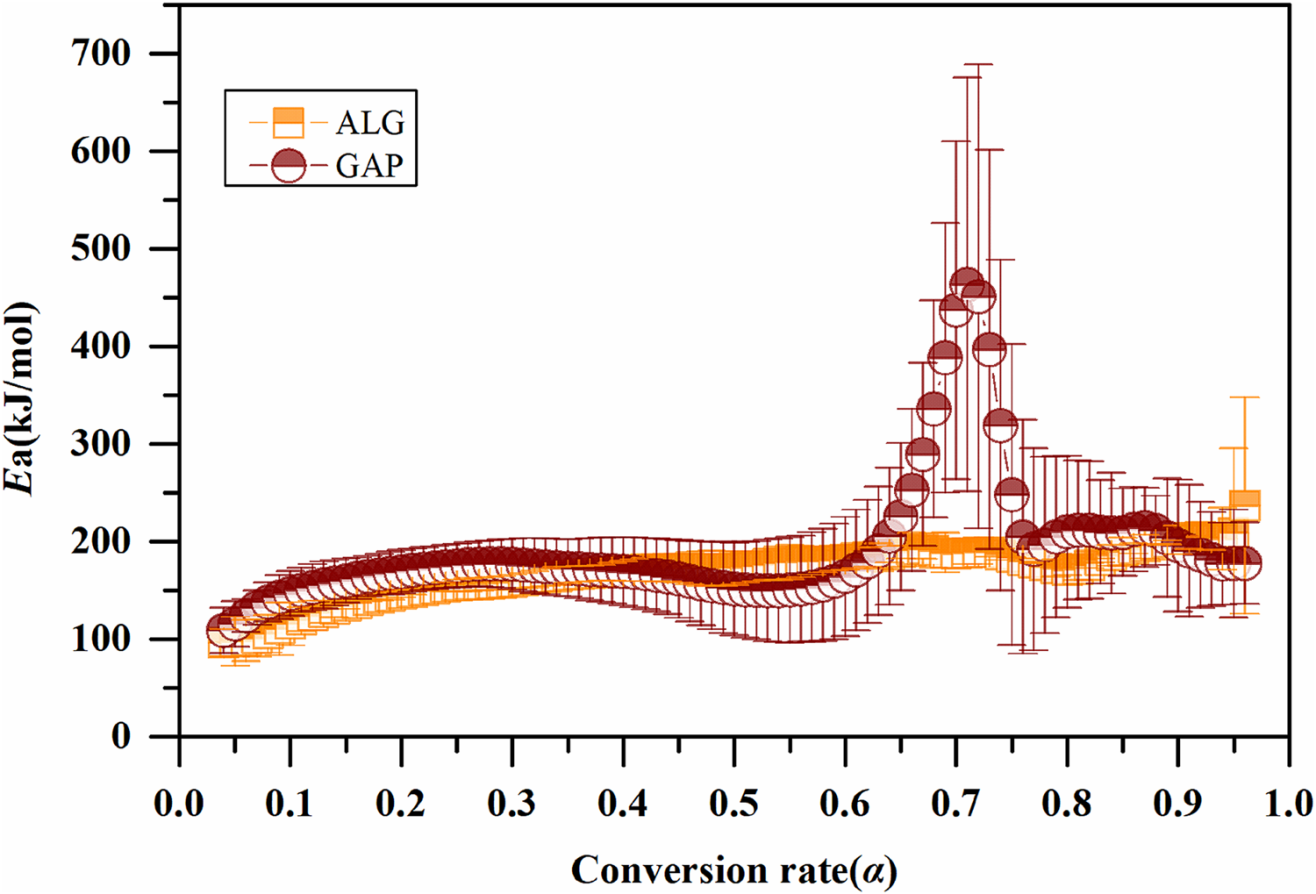
Activation energy profiles of ALG and GAP over conversion rate.

To study the interaction between the GAP and the Al particles in the first stage, Friedman model free method was employed to analyze the thermodynamics parameters of the ALG particles and the GAP, and results are shown in Fig. 3. With the increase of the conversion rate (*α*) from 0.04 to 0.6, the activation energy (*E*a) of the ALG particles or the GAP both kept within the range of 90–180 kJ mol^−1^. However, with the increase of the *α* from 0.6 to 0.7, the *E*a of the GAP instantly increased by 280 kJ mol^−1^, while that of the ALG particles maintained at the same range as before. We could learn from the TGA thermograph that, the temperature at which *α* = 0.7, corresponded to the onset temperature of the second stage of weight loss of the GAP. The *E*a of the ALG particles significantly lower than that of the pure GAP indicated the Al particles could promote the second stage decomposition of the GAP.

The second process ranged from 404 to 677 °C corresponded to the oxidation of the GAP carbonaceous residue and the Al particles^31,32^. This conclusion was reached based on the DSC thermographs of GAP and nAl in Fig. 2a: the oxidation process of the GAP carbonaceous residue and the Al particles separately ranged from 404 to 677 °C and from 500 to 660 °C^31,32^. In the DSC thermograph of the ALG, the exothermic peak of the GAP carbonaceous residue was not obvious because the amount of the residue was small. As for the oxidation process of the Al particles, the heat release of the ALG particles (2191 J g^−1^) was lower than that of the nAl particles (2706 J g^−1^). However, the sum heat release of the two stages for the ALG particles was comparable to that for the nAl particles. Besides, the onset oxidation temperature (*T*_on_) and the peak temperature of the ALG particles were separately 11 °C and 17 °C lower than those of the nAl particles, indicating the ALG particles was more active than the nAl particles. It might be caused by two reasons: ① the early oxidation of the GAP carbonaceous residue activated the oxidation of the Al particles; ② the heat and mass transfer to the active Al particles was much faster as the protection of GAP led to thinner oxide layer.

To further investigate the impact of the GAP layer on the oxidation of the Al particles, the thermal reaction integral model functions were fitted using non-isothermal chemical reaction dynamics. The *E*a and the pre-exponential factor (*A*) were calculated by Ozawa method, and 41 types of kinetic model functions and *α*-*T* data were calculated. Eventually, the most probable mechanism functions and kinetics parameters were selected and shown in Table 1.

**Table 1:** Kinetic parameters of exothermic reactions of the ALG and the nAl.

Sample	*Ea*/(kJ mol^−1^)	lg(*A*/s^−1^)	*R*	The most possible functions	Kinetic equation
ALG	442.74	25.17	0.982	$\frac{2}{3}(1 - \alpha )^{\frac{2}{3}} [1 - (1 - \alpha )^{\frac{1}{3}} ]^{ - 1}$	$$\begin{gathered} 10^{24.99} \times (1 - \alpha )^{\frac{2}{3}} [1 - (1 - \alpha )^{\frac{1}{3}} ]^{ - 1} \hfill \\ \times e^{ - 53252.3/T} \hfill \\ \end{gathered}$$
nAl	458.02	25.57	0.963	$\frac{1}{3}(1 - \alpha )[ - \ln (1 - \alpha )]^{ - 2}$	$$\begin{gathered} 10^{25.09} \times (1 - \alpha )[ - \ln (1 - \alpha )]^{ - 2} \hfill \\ \times e^{ - 55090.2/T} \hfill \\ \end{gathered}$$

As can be seen from Table 1 that the mechanism of the ALG oxidation process conformed to the Jander equation of n = 2, and the process was controlled by the three-dimensional diffusion rate. The integral formula was *G*(*α*) = [1 − (1 − *α*)^1/3^]^2^, and the corresponding differential form was *f*(*α*) = 2/3(1 − *α*)^2/3^[1 − (1 − *α*)^1/3^]^−1^. At the moment, the oxidation of the Al particles and GAP carbonaceous residue simultaneously proceeded. The Al particles were surrounded by hot air and the oxide products of GAP. The oxidation rate of the Al particles relied on the diffusion rate of these oxidizing gases. Therefore, the most probable mechanism function was differential equation *f*(*α*). By substituting the *E*a and *A* into the equation d*α*/d*t* = *Af*(*α*)e^−*E*/*RT*^, the kinetic equation could be acquired as d*α*/d*t* = 10^24.99^ × (1 − *α*)^2/3^[1 − (1 − *α*)^1/3^]^−1^ × e^−53252.3/*T*^.

The oxidation process of the nAl particles was controlled by the third order Avrami-Erofeev mechanism. The integral formula was *G*(*α*) = [− ln( 1 − *α*)]^3^, and the corresponding differential form was *f*(*α*) = 1/3(1 − *α*)[− ln(1 − *α*)]^−2^. This was because the nAl particles had oxide shells on the surface. The reaction rate of the Al particles was governed by the growth rate of alumina. The data in the Table 1 showed that the apparent activation energy of the ALG particles was 15.28 kJ mol^−1^ lower than that of the nAl particles (Ozawa method). This was because the oxidation process of the GAP and the Al particles were in the same temperature range, and under such condition, the disappearance of the GAP accompanied with the generation of the oxide shell. Therefore, the adjacent GAP could directly supply spacious oxidizing gases to the Al particles without dense shell barrier, which would enhance the gas transfer to the inside Al particles and benefit for the deep oxidation of Al particles.

### FTIR spectra of reaction products

**Figure 4:**
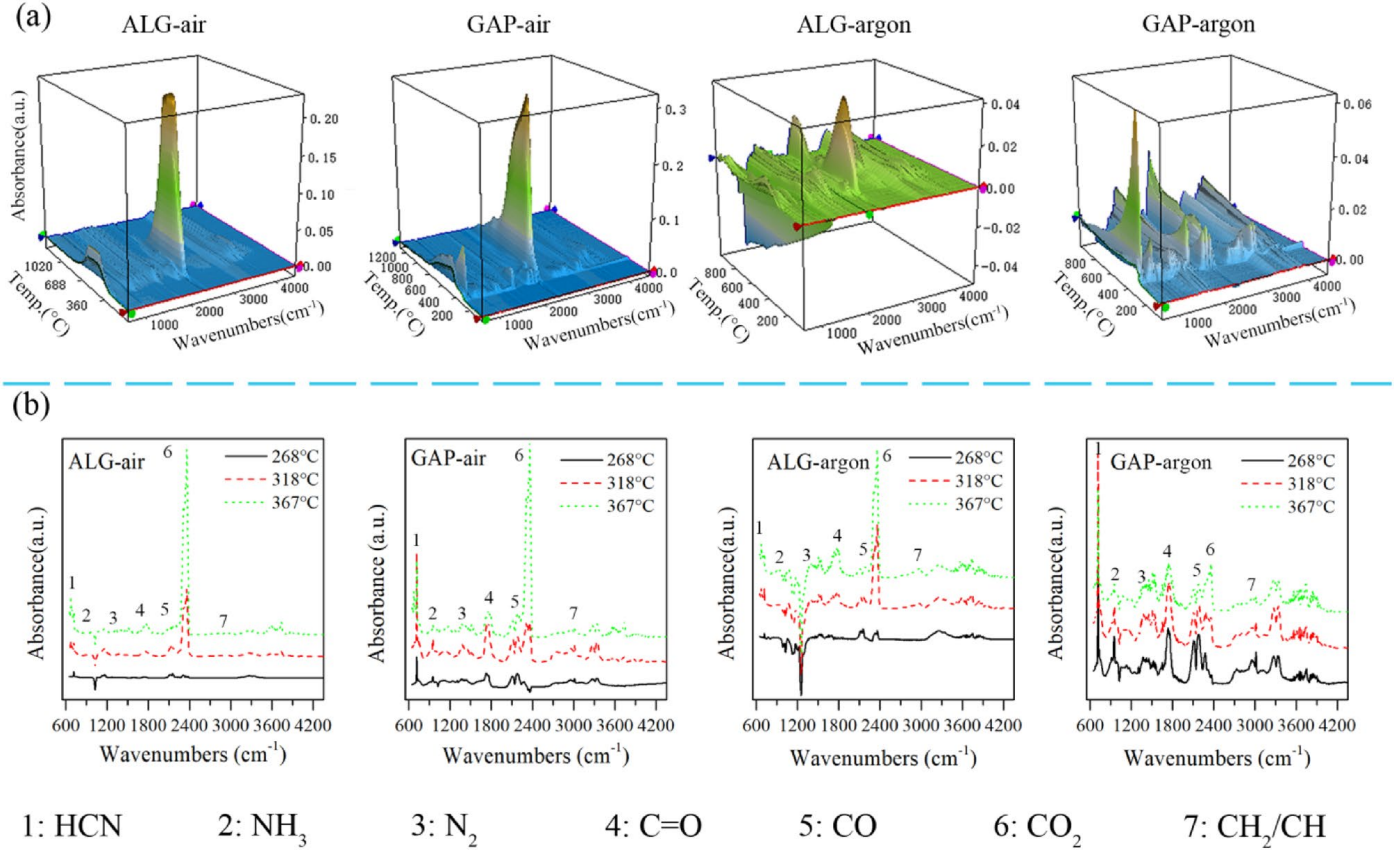
3-D FTIR spectra (**a**) and the corresponding FTIR spectra (**b**) of decomposition products of ALG and GAP in air and argon atmospheres.

To understand reaction route of the ALG particles in air, the FTIR spectra of its reaction products were detected, as shown in Fig. 4. To make a comparison, the FTIR spectra of the reaction products of the ALG in argon, and of the GAP in air and argon are also shown in Fig. 4.

Figure 4a displayed the 3-D FTIR spectra of the reaction products of the ALG particles and the GAP in air and argon. From the 3-D FTIR spectra, we could get the general evolvement of the products with the increase of temperature. In general, the 3-D FTIR spectrum of the ALG particles was similar to that of the GAP in the air atmosphere, and they both had one prominent peak at around 2000 cm^−1^. However, their spectra in argon were different from each other: the peak with strongest intensity was located at around 2000 cm^−1^ for the ALG, but at around 1000 cm^−1^ for the GAP.

In order to better identify the products, spectra at temperatures of 286 °C, 318 °C and 367 °C were extracted and displayed in Fig. 4b. Seven reaction products were observed at 367 °C for both samples: HCN (713 cm^−1^), NH_3_ (950 cm^−1^), N_2_ (1357 cm^−1^ and 2100 cm^−1^), C=O (1742 cm^−1^), CO (2114 cm^−1^ and 2180 cm^−1^), CO_2_ (2360 cm^−1^), and CH_2_/CH (3039 cm^−1^)^33–37^. The seven products except CO_2_ and HCN went through the same evolving trend in both atmospheres. In air atmosphere, the intensity of CO_2_ peak was stronger than that of HCN from 269 to 367 °C in the case of ALG; while the intensity of CO_2_ peak was not stronger than that of HCN until 367 °C in the case of GAP. Besides, under the argon environment, the intensity of CO_2_ was stronger than that of HCN for the ALG, on the contrary, the intensity of CO_2_ was weaker than that of HCN for the GAP. Based on the above analyses, it could be concluded that the interaction between the GAP and the Al particles would change the decomposition route of the GAP to end up with more oxycarbide products rather than carbonitride products.

### MS spectra of reaction products

**Figure 5:**
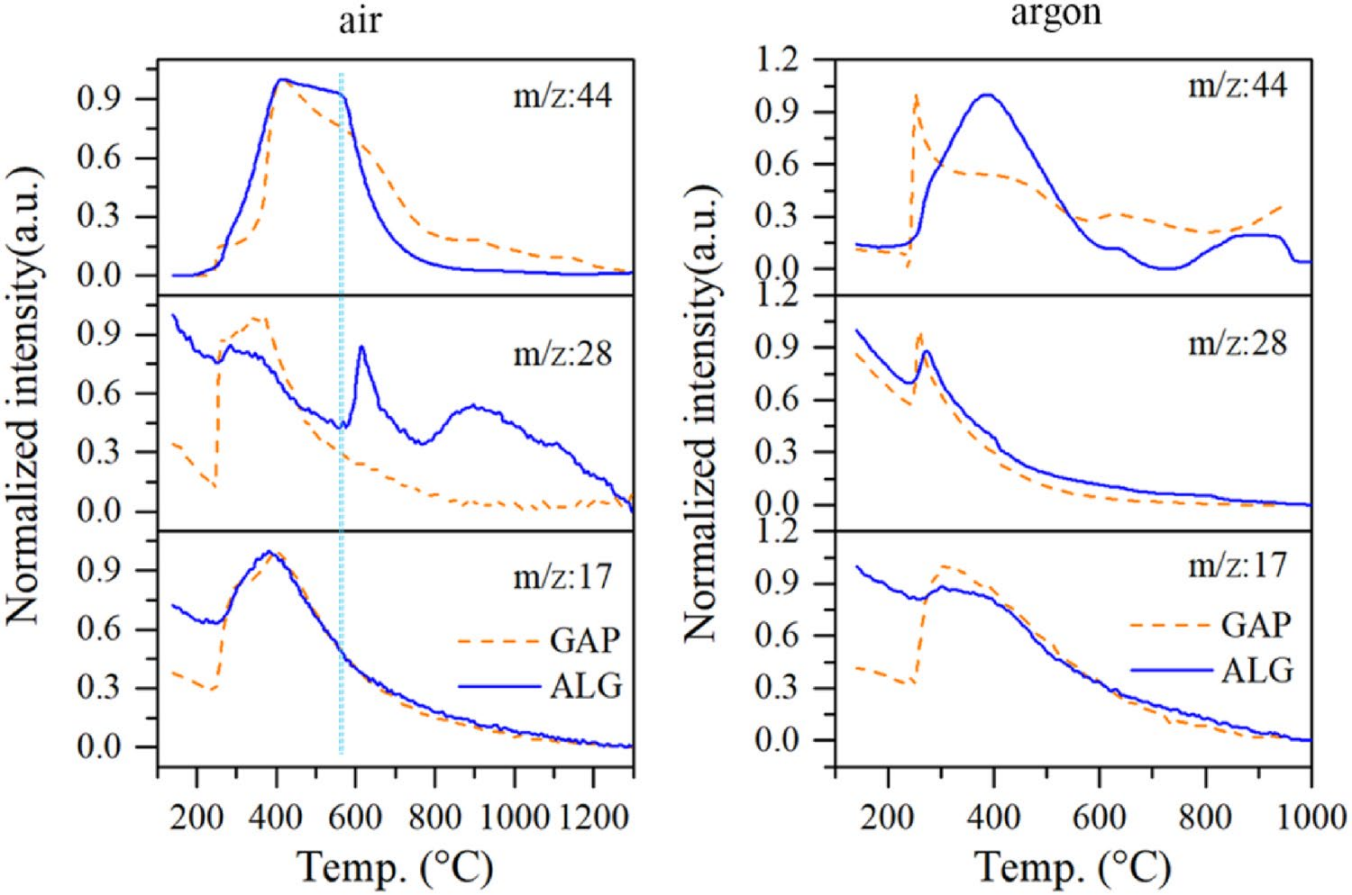
MS spectra of decomposition products of ALG and GAP in air and argon atmospheres.

MS spectra of reaction products of the ALG in air were simultaneously recorded, and compared with that of the ALG in argon and those of the GAP in air and argon. Since abundances of mass to charge ratios (m/z) of 17, 28, and 44 were not small in the spectrum of ALG, a detailed investigation had been made of these signals, as shown in Fig. 5. Among the three species, only the species at m/z = 17 exhibited the same evolving behavior for ALG and GAP in the same atmosphere. According to the FTIR spectra and the work of^34^, the species at m/z = 17 should be NH_3_, which was generated by the decomposition of intermediate product RCH = NH. The same evolving behavior of NH_3_ suggested this decomposition route of the GAP was not affected by Al particles.

**Figure 6:**
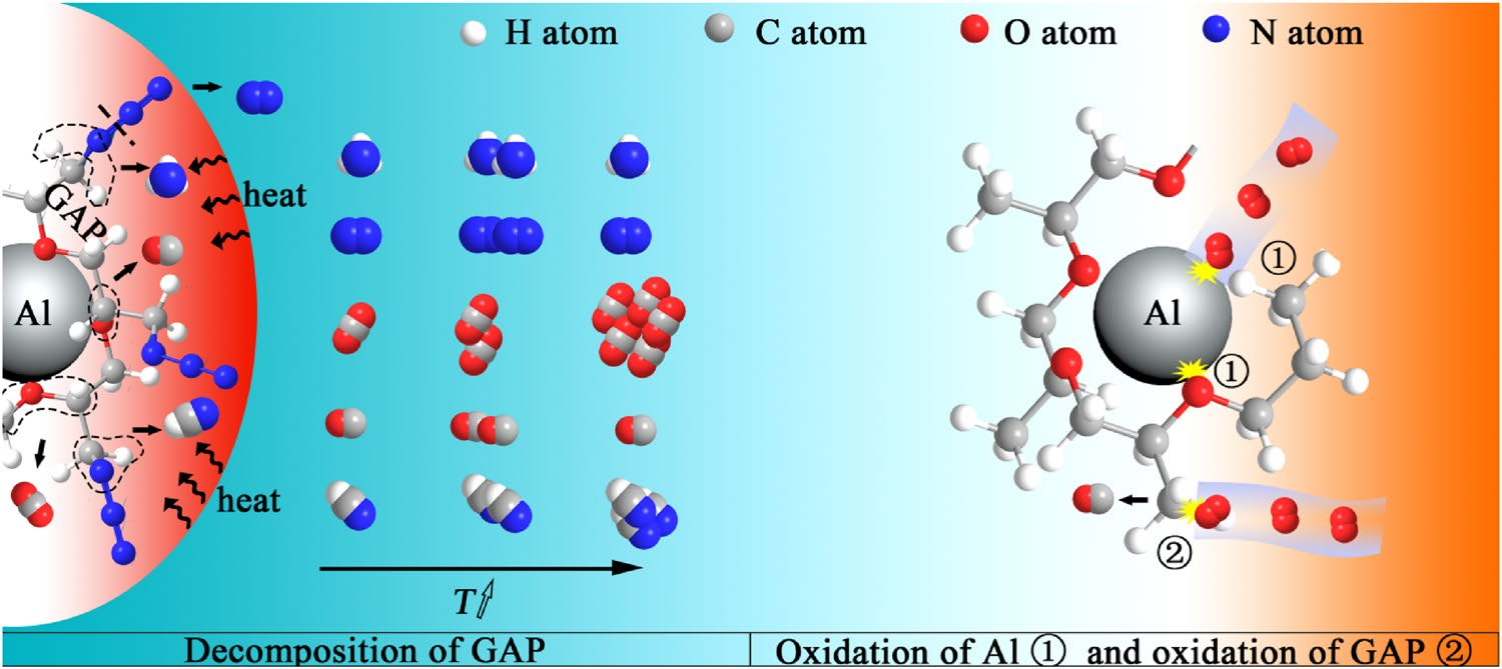
Reaction mechanism illustration of the ALG particles in air.

The species at m/z = 28 for the ALG in air exhibited the same trend to the GAP before 550 °C. According to the FTIR spectra and previous studies^35,36,38–40^, the species at m/z = 28 before 550 °C was composed of N_2_ and CO. The species at m/z = 28 of the ALG in air emerged one more peak after 550 °C. And the emergence of the new peak was accompanied by the disappearance of the species at m/z = 44 (CO_2_). Therefore, it could be inferred that the species at m/z = 28 was CO. The generation of the CO was because the oxidizing gas preferentially supplied to the Al particles, and no enough oxidizing source for the generation of CO_2._ This suggested the decomposition products of the GAP could supply oxidizing source to the Al particles, which would speed up and deepen the oxidation of the Al particles. Furthermore, in argon atmosphere, the peak of CO_2_ for the ALG particles reached to the strongest intensity at 400 °C, which was 150 °C higher than the case of the GAP. This delay generation of CO_2_ suggested that oxygen atoms of the GAP were more prone to bond with the Al atoms than with the carbon atoms of the GAP due to the intense interaction between the Al particles and the GAP. The reaction mechanism of ALG in air was therefore concluded as such in Fig. 6.

### *P–t* test

**Figure 7:**
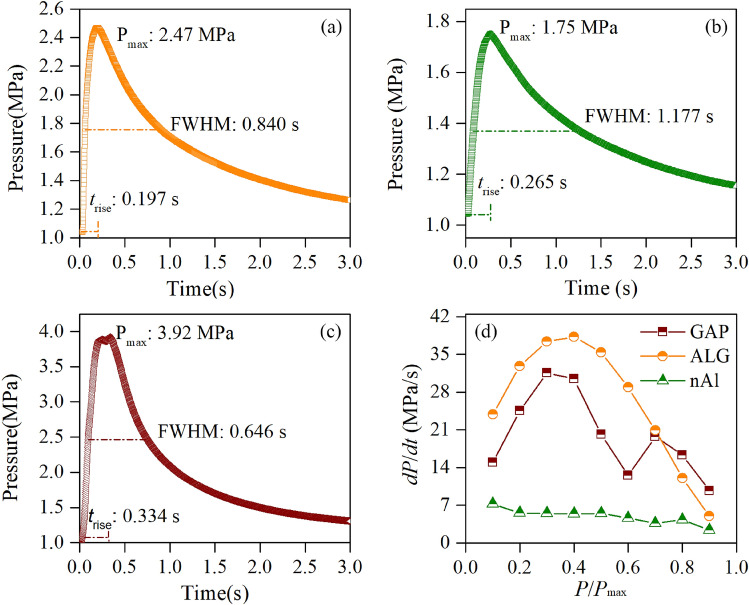
*P*–t curves of (**a**) ALG, (**b**) nAl and (**c**) GAP under 1 MPa oxygen environment; and their corresponding pressure evolvement histories (**d**).

To evaluate the energy output potential of the ALG particles, a *P*–*t* (pressure–time) test for the ALG particles was conducted, and as a comparison, *P*–*t* tests for the nAl particles and the GAP were also conducted. The *P*–*t* test results are shown in Fig. 7. When it came to the evaluation of the energy output, three parameters in the *P*–*t* curve mattered most: maximum pressure (*P*_max_), full width at half maxima (FWHM) and rise time (*t*_rise_). The *P*_max_ was determined by the instant heat release and the volume of gaseous products. The FWHM indicated the duration of the heat release process. The *t*_rise_ was used to estimate the reaction rate: the shorter the *t*_rise_ the faster the reaction proceeded.

Figure 7a–c displayed the *P*–*t* curves of the ALG particles, the nAl particles and the GAP, respectively. The *P*_max_ of the ALG particles, the nAl particles and the GAP were separately 2.47 MPa, 1.75 MPa, and 3.92 MPa. The *P*_max_ of the ALG particles was 36% smaller than that of the GAP because the amount of the gas products of the former was much less than that of the latter. The *P*_max_ of the ALG was 41% larger than that of the nAl, due to the additional heat release and gas products generated by the decomposition of the GAP coating layer. Besides, the *t*_rise_ and the FWHM of the ALG were separately 0.197 s and 0.840 s, which were separately decreased by 26% and 29% compared to those of the nAl particles. It indicated the reaction rate of the former was faster than that of the latter, which was consistent with the DSC results. To further understand the evolvements of the pressures before reaching to the *P*_max_, the d*P*/d*t* histories over *P*/*P*_max_ of the three samples were analyzed, as shown in Fig. 7d. For the case of the ALG particles, the d*P*/d*t* kept increasing with the increase of *P*/*P*_max_ before *P*/*P*_max_ = 0.4. However, for the case of the nAl particles, the d*P*/d*t* simply decreased with the increase of the *P*/*P*_max_. Furthermore, the d*P*/d*t* of the ALG particles was larger than that of the nAl particles from *P*/*P*_max_ = 0.1 to *P*/*P*_max_ = 0.9; In addition, the d*P*/d*t* of the ALG particles was larger that of the GAP before *P*/*P*_max_ = 0.7. These phenomena indicated the high pressure of the ALG particles was not caused by the simple addition of the decomposition of the GAP and the oxidation of the Al particles, but by the synergistic effect between the GAP and the Al particles.

### LASEM experiment

**Figure 8:**
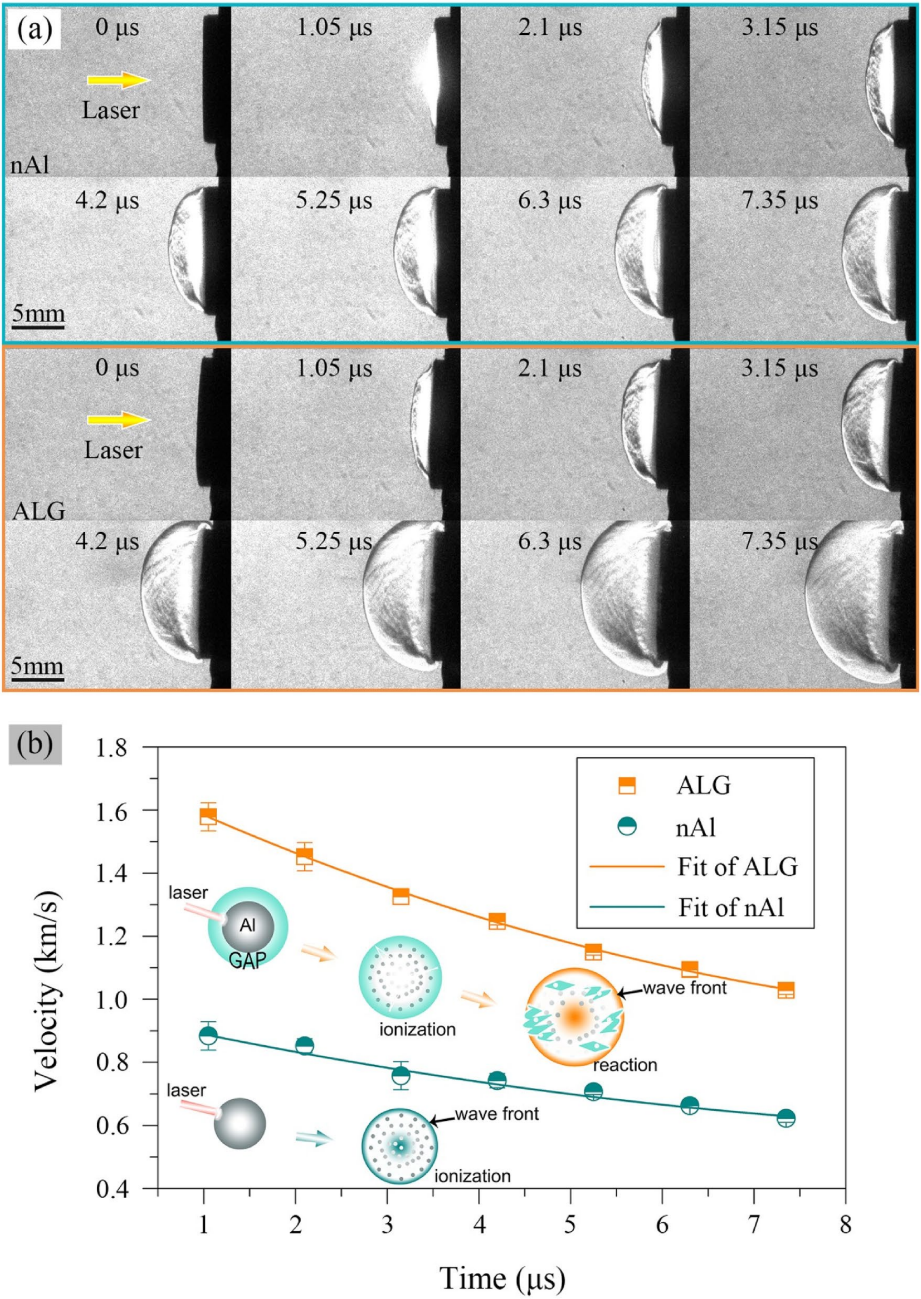
Schlieren images shock wave generated by nAl and ALG under laser stimulus (**a**) and wave front velocity (y-intercept of a 2nd order polynomial fit) (**b**).

A series of schlieren images of the shock waves of the nAl particles and the ALG particles are shown in Fig. 8a. The velocity propagation histories of the shock wave fronts were deduced from these images, as shown in Fig. 8b. Here the position of the “front” meant the fronts on the laser incident axis. Due to the limitation of the camera, only 8 frames could be got at each shot. Therefore, characteristic shock wave velocity for each sample was calculated with time range up to 7.35 μs.

As shown in Fig. 8b, the velocity vs. time fitted to a 2nd order of polynomial with quality of the fit more than 0.99. The shock wave velocity of the ALG particles was larger than that of the nAl particles from 1.05 to 7.35 μs. With the time increasing, the velocities of the shock waves for both samples gradually decreased due to the energy loss by overcoming the resistance of surrounding atmosphere. The largest velocity of the shock wave of the nAl particles was around 0.9 km s^−1^, which was comparable to the value in the work of Davari^41^. The largest velocity of the shock wave of the ALG particles could reach to 1.6 km s^−1^, which was 0.7 km s^−1^ larger than that of the nAl particles. A material that produced a faster laser-induced shock velocity was indicative of a material with a faster energy release rate^13^. Based on the above analyses, we could infer that the ALG particles owned enhanced energy release rate over the nAl particles.

In the experiment condition, the laser fluence work on the sample was as large as 0.314 GW cm^−2^. Under such laser fluence, the Al particles would be ionized and form a high temperature and pressure environment which lead to the propagation of shock wave^42^. In the case of the nAl particles, the shock wave was supported only by the energy from the ionization of the Al particles. The ALG particles maintained higher shock wave velocity than the nAl particles, suggesting additional energy was generated to support the propagation of the shock wave in addition to the ionization energy of the Al particles. This indicated the GAP coating strategy could retain the energy content of Al particles.

In summary, we demonstrated in-situ coating Al nanoparticles with nitrogen-rich binder (GAP) could result in composite particles with enhanced reactivity and energy content. By facile in-situ coating technique, the GAP could homogeously coated on the Al particles. The GAP coating could lower the onset oxidation temperature of Al particles and accelerate the oxidation of the Al particles. Reciprocally, the Al particles were able to facilitate the second stage decomposition of the GAP, and made the GAP decompose into more oxycarbide species than carbonitride species. In addition, the *P*–*t* test showed the ALG particles owned higher peak pressure and faster pressurization rate than the nAl particles. Furthermore, the LASEM experiment indicated the energy release rate of the ALG particles was faster than that of the nAl particles. In conclusion, with our coating strategy, the energy content as well as the activity of the Al particles could be well preserved by the GAP coating layer.

## Methods

### Materials

The heptane (AR) and the ethyl acetate (AR) were both purchased from Sinopharm Chemical Reagent Co., Ltd. The GAP (Mn ≈ 3280 g mol^−1^, hydroxyl value: 0.513 mmol g^−1^) was purchased from Liming Research &Design Institute of Chemical Industry Co., Ltd. The Al wire was purchased from Shijiazhuang Zhongli Zinc Industry Company.

### Sample preparation

The Al nanoparticles were produced by electrical explosion of wire (EEW) technique. To ensure the safety and protect the Al from oxidation, the explosion chamber was subjected to a constant flow of argon to maintain an inert environment. The default storage capacitance of the machine was 170 μF, and the voltage prior to discharge was arranged to be 1.2 kV. After the first explosion, the capacitor and the Al wire would be recharged and fed automatically to start another explosion. An Al wire with 0.2 mm in diameter and 48 mm in length was exploded. The metal purity of the Al wire was 99.5%. After the fabrication process, the Al nanoparticles was collected and transferred to the flask with the heptane under the protection of argon.

**Figure 9:**
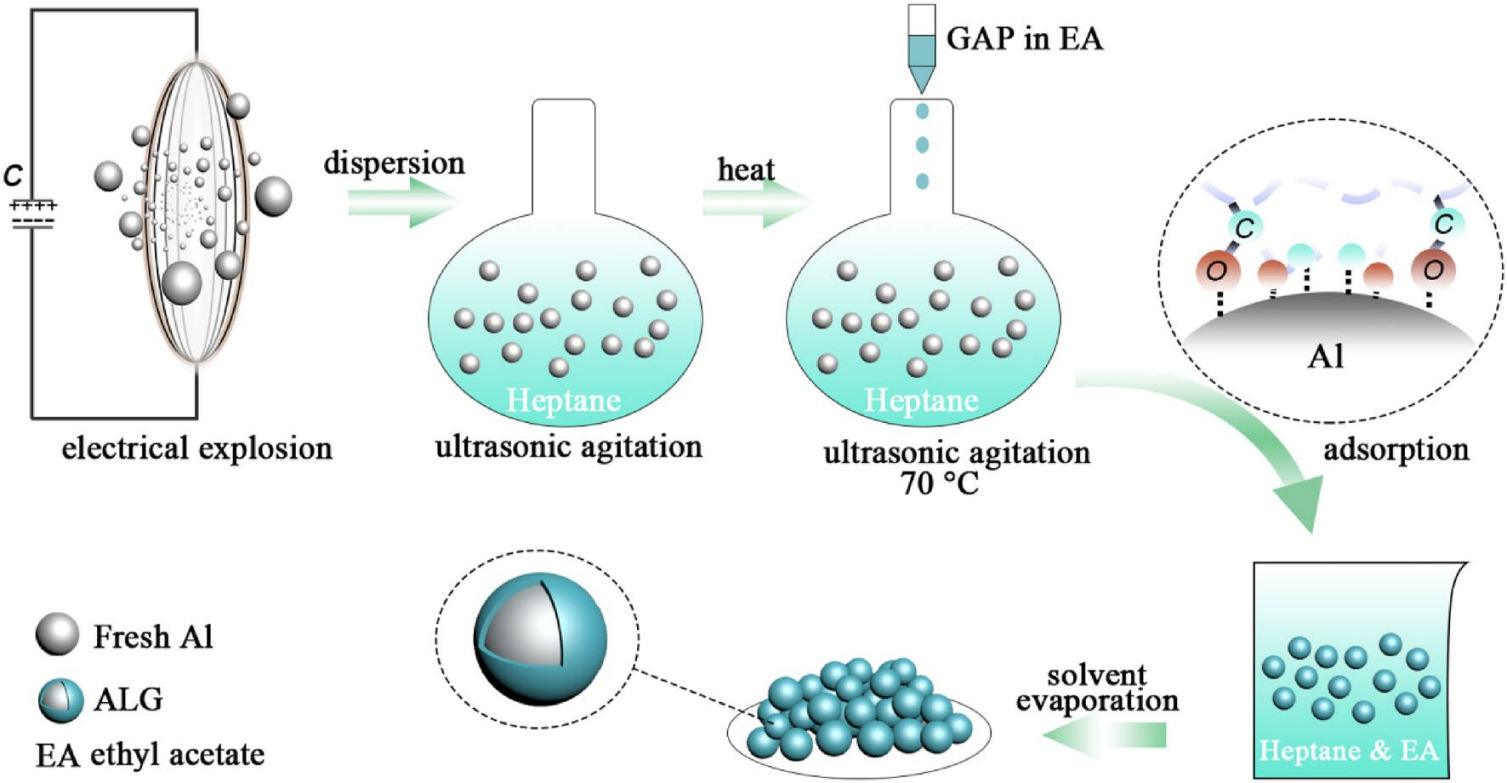
Diagram of ALG preparation process.

For the coating preparation, the ethyl acetate was used to dissolve the GAP at 70 °C under ultrasonic agitation. To ensure the stability of the GAP solution during the coating process, the Al blend was also kept at 70 °C. The GAP solution was added dropwise into the Al blend under agitation. The diagram of preparation process is shown in Fig. 9. The resulted mixture was naturally cooled down to 40 °C and kept that way until the solvent was fully evaporated. Following that, the product was vacuum dried in the furnace at 70 °C for 24 h. The product was identified as ALG. The GAP content in ALG was 16.7%. To make a comparison, the Al nanoparticles (nAl) without coating were prepared under the same condition.

### Sample characterization

The morphology of the ALG particles was observed by a scanning electron microscope (SEM, S-4800, Hitachi, USA) and a transmission electron microscope (TEM, Tecnai G2 F20, FEI Co., USA). The size distribution of the ALG particles was characterized by a laser granularity analyzer (NANOTRAC FLEX, Microtrac MRB, Germany). The localized nanoscale mid-IR measurement was carried on a Nano-IR2 system (AFM-IR, Anasys Instruments, CA, USA) over the range of 900–1500 cm^−1^. In the mid-IR measurement, the tapping-mode atomic force microscope (AFM) tip was used to interact with the sample. The resonance frequency of the tapping tip was approximately 75 kHz. By scanning the surface, an AFM tip could get the morphological and optical state of the sample due to the near-field interaction between the tip and the sample. The infrared spectrum was obtained by a VERTEX 70 Fourier Transform infrared spectroscopy (FTIR; Bruker, Germany) over the range of 400–4000 cm^−1^.

### Thermal analysis by simultaneous DSC-TGA-MS-FTIR

To reveal the decomposition mechanism, the differential scanning calorimetry (DSC), thermal gravimetric analysis (TGA), coupled with mass spectrometry (MS) and Fourier Transform infrared spectroscopy (FTIR) were performed by NETZSCH STA 449 F3 (NETZSCH, Co., Germany), NETZSCH QMS 403C (NETZSCH Co., Germany) and Bruker Vertex 70 (Bruker Scientific Instruments, USA), respectively. The DSC-TGA-MS-FTIR tests were conducted at heating rate of 10 °C min^−1^ under the argon or the air flow of 50 mL min^−1^, respectively.

### *P*–*t* test

The *P*–*t* test was conducted in a confined-volume chamber of 330 mL under oxygen environment of 1 MPa. In each test, the sample weight was kept at 0.5 ± 0.005 g. Samples were ignited by nichrome wire with 0.2 mm in diameter and 90 mm in length. The supplied voltage was 24 V. The measurements were conducted under the environment temperature of 25 °C.

### LASEM experiment

**Figure 10:**
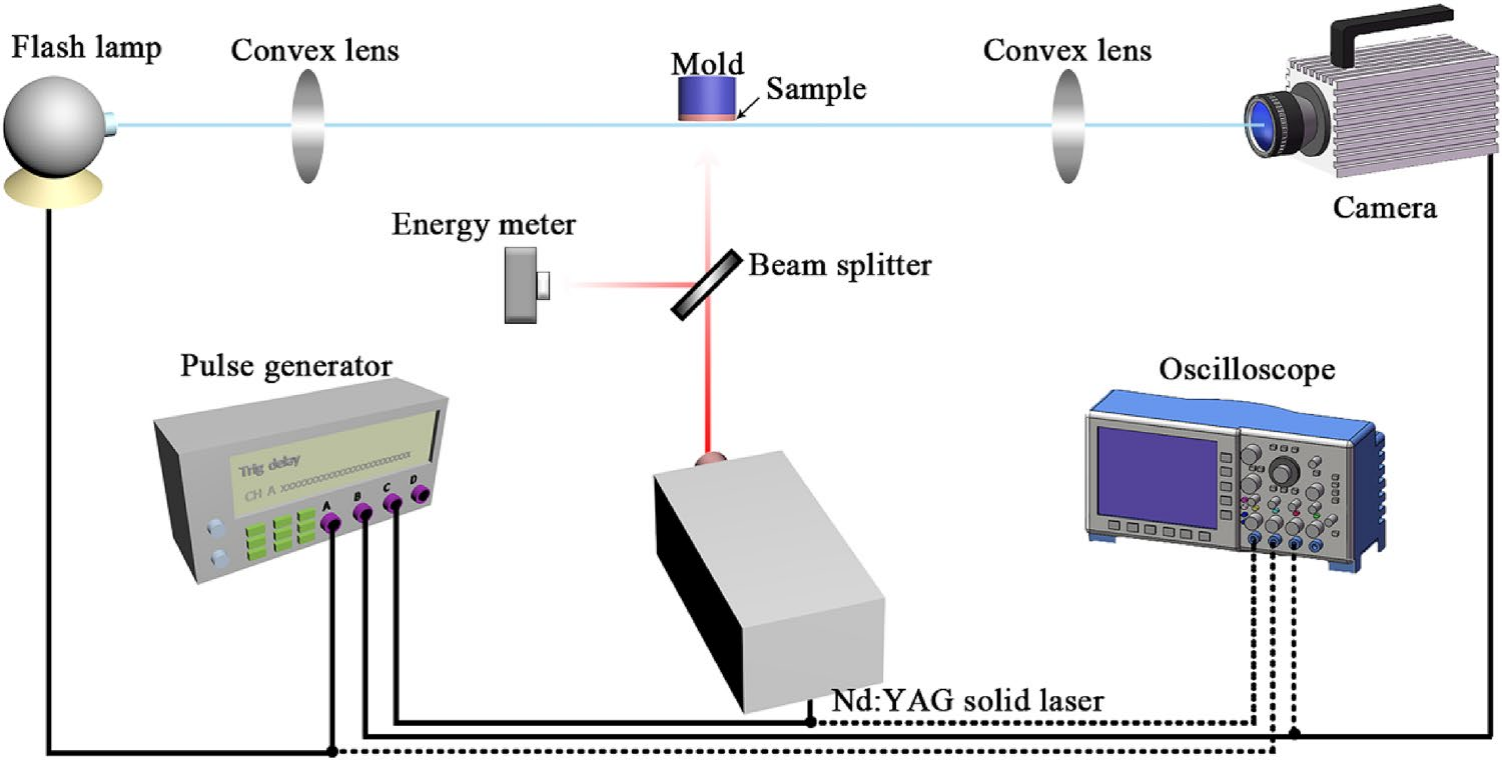
Diagram of experiment setup for the reaction under laser stimulus.

A Nd:YAG laser (1064 nm, InnoLas Laser, Germany) with maximum energy around 1030 mJ was applied to ignite the sample. The duration of each laser pulse was 6 ns. The diameter of the spot was 6 mm at wavelength of 1064 nm. In the experiment, a schlieren system was used to detect the density changes of the air caused by the reaction. The density change was recorded by a high speed camera (SIMD8, Specialized Imaging Ltd, UK) in the rate of two millions fps. A flash lamp system (JML-C2, Germany) was used to serve as the backlight with a pulse duration ranged from 0.5 to 1.2 ms. The system was triggered by a pulse generator (DG535, Stanford Research Systems, Inc., USA) under the precise time delay measured by oscilloscope (DPO7104, Tektronix, USA). An energy meter (J-50 MB-YAG, Coherent, USA) was used to measure the energy output of the laser. In each test, one laser pulse of 534 mJ was applied to the sample. The samples were stuck to the mold surface with the help of a dual adhesive tape. The experiment setup is shown in Fig. 10.
